# Application of action research in the field of healthcare: a scoping review protocol

**DOI:** 10.12688/hrbopenres.13276.1

**Published:** 2021-05-07

**Authors:** Mary Casey, David Coghlan, Áine Carroll, Diarmuid Stokes, Kinley Roberts, Geralyn Hynes

**Affiliations:** 1 UCD School of Nursing, Midwifery & Health Systems, University College Dublin, Dublin 4, Ireland; 2 Trinity Business School, University of Dublin, Trinity College, Dublin 2, Ireland; 3 School of Medicine, University College Dublin, Dublin 4, Ireland; 4 National Rehabilitation Hospital, Dun Laoghaire, Co Dublin, Ireland; 5 The Library, University College Dublin, Dublin 4, Ireland; 6 School of Nursing & Midwifery, University of Dublin, Trinity College, Dublin 2, Ireland

**Keywords:** Action research, quality in action research, action research in healthcare, participation

## Abstract

**Background: **Traditional research approaches are increasingly challenged in healthcare contexts as they produce abstract thinking rather than practical application. In this regard, action research is a growing area of popularity and interest, essentially because of its dual focus on theory and action. However, there is a need for action researchers not only to justify their research approach but also to demonstrate the quality of their empirical studies. Therefore, the authors set out to examine the current status of the quality of extant action research studies in healthcare to encourage improved scholarship in this area. The aim of this scoping review is to identify, explore and map the literature regarding the application of action research in either individual, group or organisational domains in any healthcare context.

**Methods:** The systematic scoping review will search the literature within the databases of CINAHL, PubMed and ABI/Inform within the recent five-year period to investigate the scientific evidence of the quality of action research studies in healthcare contexts. The review will be guided by Arksey and O'Malley’s five mandatory steps, which have been updated and published online by the Joanna Briggs Institute. The review will follow the PRISMA-ScR framework guidelines to ensure the standard of the methodological and reporting approaches are exemplary.

**Conclusion:** This paper outlines the protocol for an exploratory scoping review to systematically and comprehensively map out the evidence as to whether action research studies demonstrate explicitly how the essential factors of a comprehensive framework of action research are upheld. The review will summarise the evidence on the quality of current action research studies in healthcare. It is anticipated that the findings will inform future action researchers in designing studies to ensure the quality of the studies is upheld.

## Introduction

The utility and versatility of action research has brought about an increase in the level of interest, application and usage of action research in a variety of healthcare contexts in the past 20 years as healthcare systems all over the world undergo transformative change. Part of this greater interest and usage relates to the fact that in this context of change, action research aims at both taking action in a particular system in response to particular forces, and therefore brings a change, and creating knowledge about that action that provides actionable knowledge for other health care organisations. Another possible explanation for the increased application of action research in healthcare is its participatory paradigm, which invites participants to be both embedded and reflexive in the creation of collaborative learning and of actionable knowledge where research is with, rather than on or for, people. Action research therefore attempts to link theory and practice, thinking and doing, achieving both practical and research objectives ( Casey & Coghlan, 2021), and therefore provides a means of improvement by narrowing the gap between researching and implementing.

A wide range of terms are used to describe action research approaches such that it is now considered as a family of approaches ( Casey *et al.*, 2018), the common approaches being appreciative inquiry, co-operative inquiry, collaborative research, participatory action research and, more recently, co-design to name a few. The action research process involves engagement in cycles of action and reflection and always involves two goals: to address a real issue and to contribute to science through the elaboration or development of theory. These are the dual imperatives of action research. The creation of actionable knowledge is the most rigorous test of knowledge creation. Action research embodies a set of principles and outlines definite steps on how to engage in the research process. These steps are cyclical and spiral in nature and iterative and some argue that two overlapping spirals of activity exist, where one spiral depicts the research activity and the other depicts the work interest ( Casey & Coghlan, 2021). This facilitates the researchers giving adequate consideration to their own learning and knowledge as well as to all the relevant issues prior to engaging in research activity. According to Reason & Bradbury (2008:4) action research “is a living, emergent process that cannot be predetermined but changes and develops as those who engage deepen their understanding of the issues to be addressed and develop their capacity as co-inquirers both individually and collectively’.

In one of his seminal articles on action research, Lewin (1947: 147-8) describes how action research begins and develops.

Planned social action (intentional change) usually emerges from a more or less vague “idea”. An objective appears in the cloudy form of a dream or a wish, which can hardly be called a goal. To become real, to be able to steer action, something has to be developed which might be called a plan... It should be noted that the development of a general plan presupposes “fact-finding” … On the basis of this fact-finding the goal is somewhat altered…Accepting a plan does not mean that all further steps are fixed by a decision; only in regard to the first step should be the decision be final. After the first action is carried out, the second step should not follow automatically. Instead it should be investigated whether the effect of the first action was actually what was expected.

Keeping a regular check on how the inquiry process is unfolding and checking for the presence of any underlying assumptions with the group is essential ( Coughlan & Coghlan, 2002).

### Participation as a core value in action research

Action research has its focus on generating solutions to practical problems and its ability to empower practitioners because of its emphasis on participation as a core strategy ( Reason, 1994) and implementation of action ( Meyer, 2000). Active participation in a research study can be more threatening than participation in the traditional designs and there are increasing calls for evidence of impact and outcome from participation and co-design ( Palmer, 2020). Participation in healthcare is rendered complex by the different lens through which different professional groups view and understand problems through different disciplinary lens while patients must engage with these against a hierarchical background. Participation has thus been described as a multivoiced process ( Hynes *et al.*, 2012). Indeed, there is an expectation that participation from participants and co-researchers increases involvement and commitment and sustainability of action research outcomes; however, the measurement of this has been inconsistent and almost absent. For this reason we have opted to look at the degree of participation that is evidenced in the empirical studies using the ladder of citizen participation ( Arnstein, 1969), which although based on citizen participation in model cities in a department of housing and urban development, can form the basis for a more enlightened conversation about the type of participation evident in the selected studies. The ladder is organised into three major positions on citizen participation along a continuum of citizen control based on the concept of ability to exercise power. The ladder has eight rungs, with the bottom two rungs representing non-participation labelled as ‘therapy’ and ‘manipulation’. The middle section is labelled ‘degrees of tokenism’ and includes three rungs called ‘informing’, ‘consultation’ and ‘placation’ in ascending order. The higher rungs indicate three degrees of citizen power ranging from ‘partnership’ at the lower level, followed by ‘delegated power’, and ‘citizen control’ as the top rung of the ladder.

### Indicating the quality of action research studies

Action researchers do not make claims “so much on the grounds of scientific rigour, as in terms of generating findings which are useful and relevant" ( Hart & Bond, 1995:13). Baskerville & Wood-Harper (1996:238) suggest that “where the change is successful, the evaluation must critically question whether the undertaken action, among the myriad routine and non-routine organisational actions, was the sole cause of success”. According to Waterman (1998:104), “the validity of action research projects does not reside in their degree to effect change but in their attempt to improve people’s lives...through voluntary participation and cooperation”. According to Ellis & Kiely (2000:87) the validity of the research is based on the degree to which the research is useful and relevant in precipitating discussion about improvement. Morrison & Lilford (2001:441) suggest the search for knowledge can be considered scientific “if it leads to the development of theories that are explanatory: telling us why things happen as they do in that domain, comprehensively applying to the whole domain, and falsifiability: giving rise, via testable hypotheses, to empirical predictions whose persistent failure counts against the theory”. They conclude action research offers explanatory theories, and that these theories can be falsified. However, they attest these theories are context dependent and hence cannot be comprehensive.

Reason & Bradbury (2001) prefer to use the term quality rather than validity in action research as a means of expressing and judging rigour. They suggest the judge for quality action research be on the basis that it develops a praxis of relational knowledge and knowledge generation reflects co-operation between the researcher and participants. These authors also ask whether the research is guided by a reflexive concern for practical outcomes and whether the process of iterative reflection as part of the change process is readily apparent. Therefore, action research must acknowledge multiple realities and a plurality of knowing evident in the inclusion of various perspectives from the participants without attempting to find an agreed common perspective. The significance of the project is also an important aspect of quality criteria and whether the project results in new developments such as sustainable change. Therefore, a connection that integrates their different forms of expertise and different initial frameworks is needed in order to generate a third framework of the local situation. Such a frame exists in the work of Shani & Pasmore (1985/2016) who suggest that the necessary evidence of the quality of their action research studies can be achieved by: i) demonstrating knowledge of the practical and academic context of the project; ii) creating participants as co-researchers; iii) enacting cycles of action and reflection as the project is being implemented and knowledge is being co-generated; and iv) generating outcomes that are both practical for the delivery of healthcare system in the project and robust for theory development about change in healthcare. A comprehensive framework of the action research process is presented by Coghlan & Shani (2018) in terms of four factors.

1. Context
  The context of the action research project refers to individual, organisational, environmental and research/consulting factors. Individual factors include ideas about the direction and collaboration can be assured. From an organisational perspective, the availability and use of resources influence of previous history, and the level of congruence between these impacts on the capability for participation. Environmental factors in the global and local economies provide the larger context in which action research takes place. An example of research factors which can have relevance relates to previous research experience and involvement a similar area or topic.

2. Quality of relationships
  The quality of relationship refers to trust, shared language, concern for each other and equality of influence between members and researchers.

3. Quality of the action research process itself
  Refers to the dual focus on both the inquiry process and the implementation process as they are being undertaken.

4. Outcomes
  The dual outcomes of action research are some level of organisational improvement and learning and the creation of actionable knowledge.

These four factors will be used for the scoping review. A scoping review is the most appropriate approach to the literature as it provides an overview of studies, clarifying concepts or contextual information ( Pollack *et al*., 2021) and it can be used to investigate research conduct ( Munn *et al*., 2018; Tricco *et al*., 2018). This aim of this scoping review is to explore whether action research studies demonstrate explicitly how the essential factors of a comprehensive framework of action research are upheld. This is a scoping protocol for this review. Our protocol includes information about the aims and objectives of the scoping review, inclusion and exclusion criteria, search strategy and data extraction.

## Protocol

### Design

The protocol for the scoping review is based on the work of Arksey & O' Malley (2005). In addition, The Preferred Reporting Items for Systematic reviews and Meta-Analysis extension for Scoping Reviews (PRISMA-ScR) ( Tricco *et al*., 2018) will guide the process. This reporting guideline is consistent with the JBI guidance for scoping reviews, ( Tricco *et al.*, 2018). These steps are:

- Stage 1 : Identifying the research question
- Stage 2: Identifying relevant studies
- Stage 3: Study selection
- Stage 4: Charting the data
- Stage 5: Collating, summarising and reporting results

### Stage 1: Identifying the research question

The review aims to identify, explore and map the literature regarding the application of action research in either individual, group or organisational domains in any healthcare context.

***Objectives*.** To identify the degree to which the core factors of a comprehensive framework of action research ( Coghlan & Shani, 2018) are manifestly addressed. The following are the key objectives of the scoping review:

1. To identify the degree to which knowledge of the practical and academic context are addressed.
2. To establish how the quality of co-researcher relationships was maintained.
3. To determine how the quality of the enactment of cycles of action and reflection in the present tense were implemented.
4. To identify how the dual outcomes of co-generated actionable knowledge are addressed.

***Review question*.** How do researchers address the core factors of a comprehensive framework of action research in healthcare?

According to Peters *et al.* (2020b), a scoping review question should include elements of the PCC mnemonic (population, concept, and context) and it will also inform inclusion and exclusion criteria and consequently the literature search strategy.

- **Population** - healthcare professionals and patients and clients who work or come into contact with health care in any context of primary, secondary or tertiary settings
- **Concept** - studies that use an action research approach in healthcare contexts.
- **Context** - any part of health service in any country that people (healthcare professionals and patients or clients) interact with.

***Inclusion and exclusion criteria*.** The inclusion and exclusion criteria for study selection are summarised in Table 1. 

**Table 1.** Inclusion and exclusion criteria for study selection.

Inclusion criteria	Exclusion criteria
English language studies as this is the main language understood and practiced by the research team.	Non-English language studies
Human studies was selected as the review is in health services research in the context of people as practitioners and patients.	Non-human studies
Empirical action research studies as these are the key focus of this scoping review to examine how current researchers address issues of quality and validity in the conduct of their action research work.	Non-empirical studies or studies that lacked information and descriptions on the core tenets of action research. This exclusion criterion was adopted because the lack of information on the entire action research process would prevent the analysis of the application of the core tenets of action research which could be achieved through data extraction.
Any healthcare context worldwide as this is the contextual focus of the scoping review.	Non-healthcare contexts

### Stage 2: Identifying relevant studies

The research team will undertake a comprehensive search of the literature within the following databases:

- CINAHL - Nursing and Allied Health (CINAHL Plus)
- PubMed – Biomedical and life sciences database
- ABI/Inform (ProQuest) – Business database

Using the three terms of population, concept, context (PCC framework) an initial search will be deployed on CINAHL Plus. This will be followed by the use of search terms to identify key text words used to address the major concepts of population (healthcare professionals and patients), concept (action research studies in healthcare), and context (any part of health service that people interact with). Alternative terms for each of the concepts will also be included. Then each search strategy will be adapted for each database (PubMed and ABI/Inform) and specific Boolean operators, truncation markers, and MeSH headings where necessary will be used. The inclusion of the expertise of a research librarian is invaluable at an early stage of completing a scoping review ( McGowan *et al.*, 2020); the research team worked with the expert university librarian in designing and refining the search strategy and will be included as part of the research team. Sample search terms for the PubMed database are outlined in Table 2.

**Table 2.** PubMed database search strategy.

PCC concept search	
**Population** - healthcare professionals and patients and clients who work or come into contact with health care in any context of primary, secondary or tertiary settings	Patient* OR inpatient* OR outpatient* OR Client* OR End User* OR Service User* OR “advanced practitioner” OR Nurse* OR Midwi* OR Physician* OR Physiotherapists OR Physical Therapist* OR psychologist* OR “Industrial Psychology” OR “Occupational Psychology” OR Doctor* OR Consultant* OR Health Services Manager* OR Minority Group* OR Geriatric* OR “Disabled people” OR “people with Disabilities” OR Pregnant OR breastfeeding OR HIV OR “Human immunodeficiency virus” OR STI OR STD OR “Sexually Transmitted Diseases” OR “Intellectual Disability” OR “Chronically ill” OR “Patients"[Mesh:NoExp] OR "Inpatients"[Mesh] OR "Outpatients"[Mesh] OR "Nurses"[Mesh] OR "Physicians"[Mesh:NoExp] OR "Cardiologists"[Mesh] OR "Endocrinologists"[Mesh] OR "General Practitioners"[Mesh] OR "Geriatricians"[Mesh] OR "Oncologists"[Mesh] OR "Physicians, Family"[Mesh] OR "Rheumatologists"[Mesh] OR "Physical Therapists"[Mesh] OR "Psychology"[Mesh:NoExp] OR "Psychology, Industrial" [Mesh] OR “Psychology, Social"[Mesh] OR "Consultants"[Mesh] OR "Minority Groups"[Mesh] OR "Disabled Persons"[Mesh] OR "Pregnant Women"[Mesh] OR "Breast Feeding"[Mesh] OR "HIV"[Mesh] OR "Sexually Transmitted Diseases"[Mesh] OR "Intellectual Disability"[Mesh]
**Concept** - studies that use an action research approach in healthcare contexts	“Action Research” OR “Appreciative Inquiry” OR “Cooperative Inquiry” OR “Co-operative Inquiry” OR “Collaborative research” OR “Participatory Action Research” OR “Organisation Development” OR “Organization Development” OR “Organizational development” OR “Organisational Development” OR “Community Development” OR Co-design
**Context** - any part of health service in any country that people (healthcare professionals and patient or clients) interact with.	“Clinical Nursing” OR “Clinical Medicine” OR “General Practice” OR “Family Practice” OR “Community Nursing” OR “Community medicine” OR “Primary Care” OR “ **Primary Health** **Care”**OR “Acute Care” OR “subacute care” OR Paediatrics OR Pediatrics OR Geriatrics OR Gerontology OR “Medication Management” OR Drug administration OR Prescribing OR Prescriptions OR “Long-term Care” OR Long term health care OR “Mental Health Services” OR Psychiatric OR “Nursing Homes” OR Rehabilitation OR Oncology OR Pain Clinic OR pain service OR Pain management OR “Cancer hospital” OR “Cancer Care” OR “Home Nursing” OR “Public Health” OR Hospital OR “Community Development” OR “Health Policy” OR ED OR “Emergency department” OR Accident and Emergency Department* OR “Emergency service” OR Emergency medical care OR Trauma Centers OR “Hospital Medicine” OR “Health Service” OR Healthcare OR “Health Care” OR Maternity OR Maternal child nursing OR Birthing Centre* OR Birthing Center* OR Health Promotion* OR “Occupational Health” OR "Clinical Medicine"[Mesh] OR "General Practice"[Mesh] OR "Community Health Nursing"[Mesh] OR "Community Medicine"[Mesh] OR "Primary Health Care"[Mesh] OR "Subacute Care"[Mesh] OR "Pediatrics"[Mesh] OR "Geriatrics"[Mesh] OR "Medication Therapy Management"[Mesh] OR "Long-Term Care"[Mesh] OR "Mental Health Services"[Mesh] OR "Psychiatric Department, Hospital"[Mesh] OR "Social Work, Psychiatric"[Mesh] OR "Nursing Homes"[Mesh] OR "Hospitals, Rehabilitation"[Mesh] OR "Oncology Service, Hospital"[Mesh] OR "Pain Clinics"[Mesh] OR "Cancer Care Facilities"[Mesh] OR "Home Nursing"[Mesh] OR "Public Health Practice"[Mesh] OR "Hospitals"[Mesh] OR "Social Planning"[Mesh:NoExp] OR "Health Policy"[Mesh] OR "Emergency Service, Hospital"[Mesh] OR "Hospital Medicine"[Mesh] OR "Health Services"[Mesh] OR "Hospitals, Maternity"[Mesh] OR "Birthing Centers"[Mesh] OR "Health Promotion"[Mesh] OR "Occupational Health Services"[Mesh]

***Key search concepts*.** The key search concepts for this study are ‘people in healthcare’ AND ‘action research’ AND ‘healthcare environment’.

### Stage 3: Study selection

Endnote 9 will be used to manage the identified studies from the three databases. All duplicates will be removed within Endnote 9. The process of screening the titles and abstracts will be undertaken by four members of the team and non-relevant studies based on the criteria will be removed with the assistance of Rayyan (an online open access screening software tool). To resolve any conflict regarding the a difference of opinion and in the ‘undecided category, one member from the other team will chair a discussion to reach a consensus agreement. To improve reliability of the reviewers, a short training programme on the use of Rayyan will be undertaken by all the researchers and a small percentage of the studies will be screened independently by each reviewer and then a comparison will be reviewed for consistency of decision-making between the members. The full text article review will be undertaken by the same researchers using the same iterative steps, with the researchers reviewing the full texts independently.

We will do a small pilot study to test the use of the criteria and these can be modified as the researchers become more familiar with a sample of the studies to determine if further information is required of if fields are not relevant and should be removed. Data will be extracted using specified criteria and evidence from this process will be presented in table format. 

### Stage 4: Charting the data

Four members of the research team will be involved in extracting the data using a charting table created by the researchers within Microsoft Excel 365 software, as suggested by Joanna Briggs Institute (JBI) ( Peters *et al.*, 2017). The extracted data will be selected and mapped according to the specified inclusion of evidence of the quality of the action research study. Using the elements identified in the PCC framework as a guide, the initial fields will include:

- Citation details (authors and year of publication)
- Study title
- Geographical location of study
- Study setting/context
- Study aims
- Methodology/design – Type of action research

- Cited action research factors
  ▪ knowledge of the practical and academic context,
  ▪ quality of co-researcher relationships,
  ▪ quality of the enactment of cycles of action and reflection in the present tense,
  ▪ the dual outcomes of co-generated actionable knowledge.

- Type of participation ( Arnstein, 1969)
  ▪ Citizen power (citizen control, delegated power, partnership)
  ▪ Tokenism (placation, consultation, informing)
  ▪ Non-participation (therapy, manipulation)

### Stage 5: Collating, summarising and reporting the results

Data will be collected using Microsoft Excel 365 software to capture relevant information for each study by the same four members of the research team and it will be available to all members via a shared drive. Studies will be mapped according to their contextual setting, geographical location, and year of publication. All authors will discuss the data prior to analysis, which will be a descriptive analysis, as recommended by Peters *et al.* (2020a). A narrative tabular report will be produced summarising the extracted data concerning the objectives and scoping review question. The PRISMA-ScR guidelines will be used for reporting the outcomes of the review ( Tricco *et al.*, 2018). Quality appraisal of the studies will not be conducted as this review aims to explore how the core factors of a comprehensive framework of action research are addressed in each study to contribute to future development and recommendations of application of action research principles in action research in healthcare. The review will consist of thematic analysis on evidence of the quality of their action research on: i) demonstrating knowledge of the practical and academic context of the project; ii) creating participants as co-researchers; iii) enacting cycles of action and reflection in the present tense as the project is being implemented and knowledge is being co-generated; and iv) generating outcomes that are both practical for the delivery of healthcare system in the project and robust for theory development about change in healthcare. Full adherence to ethical procedures in disseminating information will be undertaken by the research team. The report will be presented both orally and through publications at national and international conferences.

## Study status

At the time of publication of this protocol, preliminary database searches had commenced.

## Conclusion

This scoping review protocol has been designed in line with the latest evidence. Action research studies were carried out in diverse healthcare settings and there are many ways of undertaking action research in healthcare that consider the research purpose, aims and theoretical underpinnings. However, there is a need demonstrate the quality of the action research studies by choosing a coherent theoretical guidance provided by scholars. This will enable the transformation and impact of action research in healthcare settings to be evaluated and thereby improve the quality of action research studies in healthcare. The results extracted from this scoping review will identify how the quality element is addressed in current empirical action research studies within a recent five-year period. Based on the outcome of the review knowledge gaps and deficits will be uncovered in relation to demonstrating adherence to quality criteria when undertaking action research studies. A Quality check list for action research studies may be generated similar in format to extant reporting criteria for qualitative and quantitative studies. Findings from the review will be shared widely with healthcare personnel both locally and nationally and also through presentations and publication of the review in an open-access journal.

## Data availability

No data are associated with this article.
