# Supplementary material for: Application of action research in the field of healthcare: a scoping review protocol
Source: HRB Open Res. 2021 Jun 15;4:46. Originally published 2021 May 7. [Version 2] doi: 10.12688/hrbopenres.13276.2 (PMC8240599; doi:10.12688/hrbopenres.13276.2)
Supplement: Peer review history

# Peer review: Reviewer response for version 2

**DOI:** 10.21956/hrbopenres.14516.r29669

**Published:** 2021-06-28

**Authors:** Victor Friedman

**Affiliations:** 1 Action Research Center for Social Justice, Max Stern Yezreel Valley College, Emek Yezreel, Israel

The changes to the article are sufficient.

Is the study design appropriate for the research question?

Partly

Is the rationale for, and objectives of, the study clearly described?

Yes

Are sufficient details of the methods provided to allow replication by others?

Yes

Are the datasets clearly presented in a useable and accessible format?

Not applicable

Reviewer Expertise:

Action research, organisational learning, social inclusion, conflict transformation, action science, field theory

I confirm that I have read this submission and believe that I have an appropriate level of expertise to confirm that it is of an acceptable scientific standard.

# Response

**Published:** 2021-06-28

**Authors:** Mary Casey

**Affiliations:** UCD Nursing, Midwifery & Health Systems, Ireland

Many thanks for your considered response that has helped us to improve our publication.

 Kind Regards

 Mary

# Peer review: Reviewer response for version 1

**DOI:** 10.21956/hrbopenres.14449.r29435

**Published:** 2021-05-28

**Authors:** Victor Friedman

**Affiliations:** 1 Action Research Center for Social Justice, Max Stern Yezreel Valley College, Emek Yezreel, Israel

This paper presents a protocol for a scoping literature review of how action research in health care deals with quality. It argues for the need for such a review, which promises to provide a deeper, more nuanced, and empirically based understanding of what quality actually means in action research in the health care field. The paper reviews a small sample of the literature on quality in action research and points to a variety of criteria/factors for evaluating/generating quality. For their scoping review, the authors choose “four factors” for quality as presented by Coghlan and Shani (2018). The paper then presents the research question, the methods to be used for (1) identifying and selecting relevant studies to be reviewed, (2) charting the data, and (3) collating, summarising and reporting the results.

 The paper makes a convincing argument for the need for such a scoping review and prevents a very clear, systematic, and well though-out protocol that should generate very useful and important knowledge. 

 At the same time, I question the authors choice of a single, pre-existing framework for quality (Coghlan & Shani,2018). After presenting a number of varying approaches to quality, they write, “a connection that integrates their different forms of expertise and different initial frameworks is needed in order to generate a third framework of the local situation.” However, the authors do not actually explain how these frameworks are integrated within the Coghlan and Shani (2018) model. It seems to me that some things are missing or need to be developed a bit more:

1.  Making a specific reference to the issues of reflection/reflexivity, which are featured in the literature reviewed earlier in the paper. These are not the same processes, though they related, and are an important component of action research.
2. The Coghlan & Shani (2018) framework is very heavily oriented towards action research in organizations. Making a specific reference to the issue of “community,” which is a central domain in health care but is missing from the “Context” part of the framework. It does appear in Table 2. Regarding Table 2, I would add “Community Based (Participatory Research (CBPR or CBR)” to “Concept” (studies that use an action research approach in healthcare contexts).
3.  “Participation” appears as a separate category outside of the framework. However, participation is applied implied in the Coghlan and Shani (2018) model by “equality of influence between members and researchers” in the “quality of relationships” (factor 3). How does quality of relationships differ from participation? Perhaps participation cold be incorporated into the framework or the framework crafted to reduce redundancy.
4. I suggest that the authors take a look at the quality choice-points for action oriented research for transformation suggested by the (Bradbury et al, 2020), https://journals.sagepub.com/doi/full/10.1177/1476750320904562. )

To sum up, Coghlan & Shani (2018) provides a very good foundation on which to build the integrative model, but a bit more work needs to be done to make it integrative and more comprehensive.

 There are also a number of editing issues:

1.  The authors write: “Therefore, a connection that integrates their different forms of expertise and different initial frameworks is needed in order to generate a third framework of the local situation.” ***What is meant by “third framework”? What were the first and second frameworks? ***
2. ***The very next sentence says *** “Such a frame exists”. ***This confuses a bit more since “framework” and “frame” are not the same***
3. The authors write: “Individual factors include ideas about the direction and collaboration can be assured.” ***There is something missing in this sentence. I think it should say “ideas about how the…” but that’s up to the authors***
4. The authors write: “From an organisational perspective, the availability and use of resources influence of previous history, and the level of congruence between these impacts on the capability for participation.” There is something missing in this sentence as well. I think there needs to be a comma: “use of resources, influence of previous history and…"
5. The authors write: “Based on the outcome of the review knowledge gaps and deficits will be uncovered in relation to demonstrating adherence to quality criteria when undertaking action research studies.” I think there is a missing comma and should read: Based on the outcome of the review, knowledge gaps…

Finally, I want to raise a thought I had about the relationship between action research and academic writing that may, or may not, be relevant to this project and the protocol. Understandably, the authors exclude research that lacks “information and descriptions on the core tenets of action research”. However, as an associate editor of the *Acton Research Journal *and a frequent reviewer of action research papers, I am often struck by the difference between doing action research and writing about it for academic journals. Unlike normal research, which can be planned and controlled to a high degree, action research, by its very nature as a participative process, is emergent and responsive to changing situations, rarely actually occurring according to “plan.” Sometimes I read manuscripts that are based on quite interesting and high quality action research, but this research is not framed or presented in a way that meets academic standards. Writing up action research for academic journals is often a *post hoc* reflective process that addresses the question “What did we learn from this project? What kind of knowledge did we produce?” In my experience, many manuscripts fail because they do not adequately frame a question, connect with the relevant literature, or adequately present the data to back up their claims. All of these problems have more to with writing than with the action research itself. In this respect, I believe that this project looks not so much at the quality of action research as the quality of action research as reflected in academic writing. I am not sure how important this distinction is, if at all, but I did want to put it on the table.

 I wish the authors all the best in carrying out this important study.

Is the study design appropriate for the research question?

Partly

Is the rationale for, and objectives of, the study clearly described?

Yes

Are sufficient details of the methods provided to allow replication by others?

Yes

Are the datasets clearly presented in a useable and accessible format?

Not applicable

Reviewer Expertise:

action research, organisational learning, social inclusion, conflict transformation, action science, field theory

I confirm that I have read this submission and believe that I have an appropriate level of expertise to confirm that it is of an acceptable scientific standard, however I have significant reservations, as outlined above.

# Peer review: Reviewer response for version 1

**DOI:** 10.21956/hrbopenres.14449.r29394

**Published:** 2021-05-11

**Authors:** Brendan McCormack

**Affiliations:** 1 Centre for Person-Centred Practice Research, Queen Margaret University Edinburgh, Musselburgh, UK

Thanks for asking me to review this protocol. It is great to see this work happen and it is to be welcomed, as it is needed. Generally the protocol is really thorough and is very clear and should produce some good outcomes.

 A couple of comments:

1. The focus is interesting to me. You clearly set out what 'counts' as action research, which includes 'co-design work in healthcare' (much of which I struggle to see as research at all!) but don't include transformational and transformative research which is usually theoretically and philosophically robust. That seems odd!
2. The databases to be searched don't include any educational or social science databases. Whilst I completely appreciate that health related publications in these databases are few, they are however places that health-focused action research gets published. I think these need to be included.
3. The methods are clearly set out and are very thorough. However I found the stage 5 of the methods to be 'vague' and I am not completely sure what the processes are and how standardised they are. I think these could be further clarified.
4. The dissemination ideas lack creativity and contemporary (non-academic publication focused) methods. These should be further considered.

Well done and I wish you luck with the project.

Is the study design appropriate for the research question?

Yes

Is the rationale for, and objectives of, the study clearly described?

Yes

Are sufficient details of the methods provided to allow replication by others?

Partly

Are the datasets clearly presented in a useable and accessible format?

Yes

Reviewer Expertise:

action research. participatory research. person-centred research. nursing and healthcare research

I confirm that I have read this submission and believe that I have an appropriate level of expertise to confirm that it is of an acceptable scientific standard.
